# Using Sex to Cure the Genome

**DOI:** 10.1371/journal.pbio.1002417

**Published:** 2016-03-17

**Authors:** Eduardo P. C. Rocha

**Affiliations:** 1 Microbial Evolutionary Genomics, Institut Pasteur, Paris, France; 2 Centre National de la Recherche Scientifique (CNRS), UMR3525, Paris, France

## Abstract

The diversification of prokaryotes is accelerated by their ability to acquire DNA from other genomes. However, the underlying processes also facilitate genome infection by costly mobile genetic elements. The discovery that cells can uptake DNA by natural transformation was instrumental to the birth of molecular biology nearly a century ago. Surprisingly, a new study shows that this mechanism could efficiently cure the genome of mobile elements acquired through previous sexual exchanges.

## Abstract 2

Natural transformation was thought to provide new genetic information to bacteria. Instead, a new study suggests it cures the genome of deleterious mobile elements.

Horizontal gene transfer (HGT) is a key contributor to the genetic diversification of prokaryotes [1]. Its frequency in natural populations is very high, leading to species’ gene repertoires with relatively few ubiquitous (core) genes and many low-frequency genes (present in a small proportion of individuals). The latter are responsible for much of the phenotypic diversity observed in prokaryotic species and are often encoded in mobile genetic elements that spread between individual genomes as costly molecular parasites. Hence, HGT of interesting traits is often carried by expensive vehicles.

The net fitness gain of horizontal gene transfer depends on the genetic background of the new host, the acquired traits, the fitness cost of the mobile element, and the ecological context [2]. A study published in this issue of *PLOS Biology* [3] proposes that a mechanism originally thought to favor the acquisition of novel DNA—natural transformation—might actually allow prokaryotes to clean their genome of mobile genetic elements.

Natural transformation allows the uptake of environmental DNA into the cell (Fig 1). It differs markedly from the other major mechanisms of HGT by depending exclusively on the recipient cell, which controls the expression of the transformation machinery and favors exchanges with closely related taxa [4]. DNA arrives at the cytoplasm in the form of small single-stranded fragments. If it is not degraded, it may integrate the genome by homologous recombination at regions of high sequence similarity (Fig 1). This results in allelic exchange between a fraction of the chromosome and the foreign DNA. Depending on the recombination mechanisms operating in the cell and on the extent of sequence similarity between the transforming DNA and the genome, alternative recombination processes may take place. Nonhomologous DNA flanked by regions of high similarity can be integrated by double homologous recombination at the edges (Fig 1E). Mechanisms mixing homologous and illegitimate recombination require less strict sequence similarity and may also integrate nonhomologous DNA in the genome [5]. Some of these processes lead to small deletions of chromosomal DNA [6]. These alternative recombination pathways allow the bacterium to lose and/or acquire novel genetic information.

**Fig 1:**
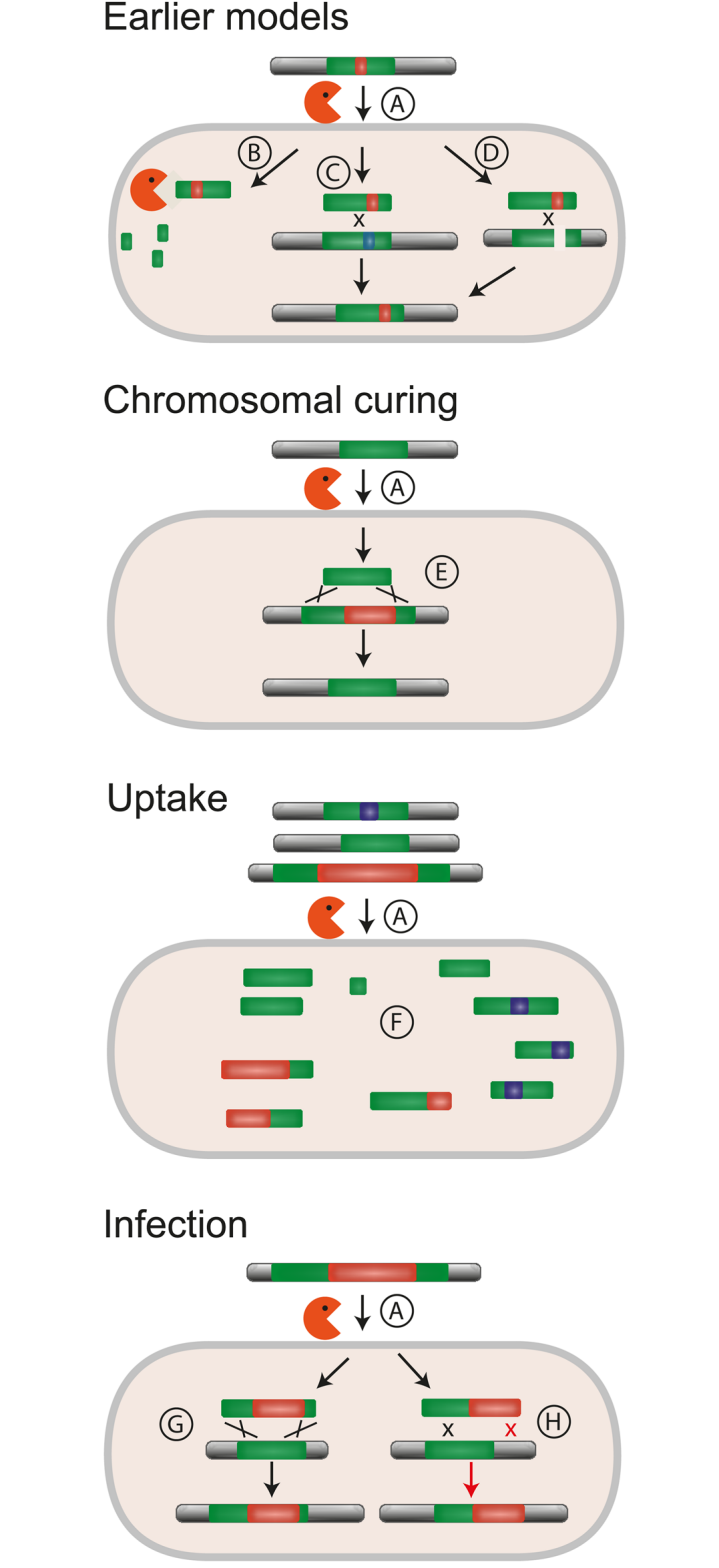
Natural transformation and its outcomes. The mechanism of environmental DNA uptake brings into the cytoplasm small single-stranded DNA fragments (A). Earlier models for the *raison d’être* of natural transformation have focused on the role of DNA as a nutrient (B), as a breaker of genetic linkage (C), or as a substrate for DNA repair (D). The chromosomal curing model allows the removal of mobile elements by recombination between conserved sequences at their extremities (E). The model is strongly affected by the size of the incoming DNA fragments, since the probability of uptake of a mobile element rapidly decreases with the size of the element and of the incoming fragments (F). This leads to a bias towards the deletion of mobile elements by recombination, especially the largest ones. In spite of this asymmetry, some mobile elements can integrate the genome via natural transformation, following homologous recombination between large regions of high sequence similarity (G) or homology-facilitated illegitimate recombination in short regions of sequence similarity (H).

Natural transformation was the first described mechanism of HGT. Its discovery, in the first half of the 20th century, was instrumental in demonstrating that DNA is the support of genetic information. This mechanism is also regularly used to genetically engineer bacteria. Researchers have thus been tantalized by the lack of any sort of consensus regarding the *raison d’être* of natural transformation.

Croucher, Fraser, and colleagues propose that the small size of recombining DNA fragments arising from transformation biases the outcome of recombination towards the deletion of chromosomal genetic material (Fig 1F). Incoming DNA carrying the core genes that flank a mobile element, but missing the element itself, can provide small DNA fragments that become templates to delete the element from the recipient genome (Fig 1E). The inverse scenario, incoming DNA carrying the core genes and a mobile element absent from the genome, is unlikely due to the mobile element being large and the recombining transformation fragments being small. Importantly, this mechanism most efficiently removes the loci at low frequency in the population because incoming DNA is more likely to lack such intervening sequences when these are rare. Invading mobile genetic elements are initially at low frequencies in populations and will be frequently deleted by this mechanism. Hence, recombination will be strongly biased towards the deletion or inactivation of large mobile elements such as phages, integrative conjugative elements, and pathogenicity islands. Simulations at a population scale show that transformation could even counteract the horizontal spread of mobile elements.

An obvious limit of natural transformation is that it can't cope with mobile genetic elements that rapidly take control of the cell, such as virulent phages, or remain extra-chromosomal, such as plasmids. Another limit of transformation is that it facilitates the acquisition of costly mobile genetic elements [7,8], especially if these are small. When these elements replicate in the genome, as is the case of transposable elements, they may become difficult to remove by subsequent events of transformation. Further work will be needed to quantify the costs associated with such infections.

Low-frequency adaptive genes might be deleted through transformation in the way proposed for mobile genetic elements. However, adaptive genes rise rapidly to high frequency in populations, becoming too frequent to be affected by transformation. Interestingly, genetic control of transformation might favor the removal of mobile elements incurring fitness costs while preserving those carrying adaptive traits [3]. Transformation could, thus, effectively cure chromosomes and other replicons of deleterious mobile genetic elements integrated in previous events of horizontal gene transfer while preserving recently acquired genes of adaptive value.

Prokaryotes encode an arsenal of immune systems to prevent infection by mobile elements and several regulatory systems to repress their expression [9]. Under the new model (henceforth named the chromosomal curing model), transformation has a key, novel position in this arsenal because it allows the expression of the incoming DNA while subsequently removing deleterious elements from the genome.

Mobile elements encode their own tools to evade the host immune systems [9]. Accordingly, they search to affect natural transformation [3]. Some mobile genetic elements integrate at, and thus inactivate, genes encoding the machineries required for DNA uptake or recombination. Other elements express nucleases that degrade exogenous DNA (precluding its uptake). These observations suggest an arms race evolutionary dynamics between the host, which uses natural transformation to cure its genome, and mobile genetic elements, which target these functions for their own protection. This gives further credibility to the hypothesis that transformation is a key player in the intra-genomic conflicts between prokaryotes and their mobile elements.

Previous studies have proposed alternative explanations for the evolution of natural transformation, including the possibility that it was caused by selection for allelic recombination and horizontal gene transfer [10], for nutrient acquisition [11], or for DNA repair [12]. The latter hypothesis has recently enjoyed regained interest following observations that DNA-damage agents induce transformation [13,14], along with intriguing suggestions that competence might be advantageous even in the absence of DNA uptake [15,16]. The hypothesis that transformation evolved to acquire nutrients has received less support in recent years.

Two key specific traits of transformation—host genetic control of the process and selection for conspecific DNA—share some resemblance with recombination processes occurring during sexual reproduction. Yet, the analogy between the two processes must be handled with care because transformation results, at best, in gene conversion of relatively small DNA fragments from another individual. The effect of sexual reproduction on genetic linkage is thought to be advantageous in the presence of genetic drift or weak and negative or fluctuating epistasis [17]. Interestingly, these conditions could frequently be met by bacterial pathogens [18], which might explain why there are so many naturally transformable bacteria among human pathogens, such as *Streptococcus pneumoniae*, *Helicobacter pylori*, *Staphylococcus aureus*, *Haemophilus influenzae*, or *Neisseria* spp. The most frequent criticism to the analogy between transformation and sexual reproduction is that environmental DNA from dead individuals is unlikely to carry better alleles than the living recipient [11]. This difficulty is circumvented in bacteria that actively export copies of their DNA to the extracellular environment. Furthermore, recent theoretical studies showed that competence could be adaptive even when the DNA originates from individuals with lower fitness alleles [19,20]. Mathematically speaking, sexual exchanges with the dead might be better than no exchanges at all.

The evaluation of the relative merits of the different models aiming to explain the *raison d’être* of natural transformation is complicated because they share several predictions. For example, the induction of competence under maladapted environments can be explained by the need for DNA repair (more DNA damage in these conditions), by selection for adaptation (through recombination or HGT), and by the chromosomal curing model because mobile elements are more active under such conditions (leading to more intense selection for their inactivation). Some of the predictions of the latter model—the rapid diversification and loss of mobile elements and their targeting of the competence machinery—can also be explained by models involving competition between mobile elements and their antagonistic association with the host. One of the great uses of mathematical models in biology resides in their ability to pinpoint the range of parameters and conditions within which each model can apply. The chromosomal curing model remains valid under broad ranges of variation of many of its key variables. This might not be the case for alternative models [3].

While further theoretical work will certainly help to specify the distinctive predictions of each model, realistic experimental evolutionary studies will be required to test them. Unfortunately, the few pioneering studies on this topic have given somewhat contradictory conclusions. Some showed that natural transformation was beneficial to bacteria adapting under suboptimal environments (e.g., in times of starvation or in stressful environments) [21,22], whereas others showed it was most beneficial under exponential growth and early stationary phase [23]. Finally, at least one study showed a negative effect of transformation on adaptation [24]. Part of these discrepancies might reveal differences between species, which express transformation under different conditions. They might also result from the low intraspecies genetic diversity in these experiments, in which case the use of more representative communities might clarify the conditions favoring transformation.

Macroevolutionary studies on natural transformation are hindered by the small number of prokaryotes known to be naturally transformable (82 species, following [25]). In itself, this poses a challenge: if transformation is adaptive, then why does it seem to be so rare? The benefits associated with deletion of mobile elements, with functional innovation, or with DNA repair seem sufficiently general to affect many bacterial species. The trade-offs between cost and benefit of transformation might lead to its selection only when mobile elements are particularly deleterious for a given species or when species face particular adaptive challenges. According to the chromosomal curing model, selection for transformation would be stronger in highly structured environments or when recombination fragments are small. There is also some evidence that we have failed to identify numerous naturally transformable prokaryotes, in which case the question above may lose part of its relevance. Many genomes encode key components of the transformation machinery, suggesting that this process might be more widespread than currently acknowledged [25]. As an illustration, the ultimate model for research in microbiology—*Escherichia coli*—has only recently been shown to be naturally transformable; the conditions leading to the expression of this trait remain unknown [26].

The chromosomal curing model might contribute to explaining other mechanisms shaping the evolution of prokaryotic genomes beyond the removal of mobile elements. Transformation-mediated deletion of genetic material, especially by homology-facilitated illegitimate recombination (Fig 1H), could remove genes involved in the mobility of the genetic elements, facilitating the co-option by the host of functions encoded by mobile genetic elements. Several recent studies have pinpointed the importance of such domestication processes in functional innovation and bacterial warfare [27]. The model might also be applicable to other mechanisms that transfer small DNA fragments between cells. These processes include gene transfer agents [28], extracellular vesicles [29], and possibly nanotubes [30]. The chromosomal curing model might help unravel their ecological and evolutionary impact.
